# Can healthcare apps and smart speakers improve the health behavior and depression of older adults? A quasi-experimental study

**DOI:** 10.3389/fdgth.2023.1117280

**Published:** 2023-02-23

**Authors:** Dasom Kim

**Affiliations:** Expert Group on Health Promotion for Seoul Metropolitan Government, Seoul, Republic of Korea

**Keywords:** aged, internet of things, information technology, depression, health behavior

## Abstract

**Purpose:**

This study identified the effects of applying information and communication technologies (ICT) to the health management of older adults aged 65 or older.

**Methods:**

Older adults registered at public health centers were provided with the health management app “Health Today” and a smart speaker for 6 months to perform assigned healthcare missions. The program was conducted for 6 months by dividing participants into two groups: one that received both the health management app and the smart speaker, and another that used only the health management app. Depression, self-efficacy, number of days of moderate-intensity exercise, relative grip strength, balance tests, and five-times-sit-to-stand tests were measured during the pre- and post-evaluation.

**Results:**

Both groups showed a positive health status and behavioral changes at post-evaluation. However, no reduced depression was observed due to communication and music listening functions in the group that was additionally provided smart speakers.

**Conclusion:**

ICT use in healthcare can be beneficial for older adults. However, whether these devices meet the purpose of the national health project must be determined, and an effect evaluation must be undertaken prior to providing these ICT devices for the health management of older adults in the public domain.

## 1. Introduction

Information and communication technology (ICT) has recently come to refer to all fields of collecting, processing, and consuming information, rather than merely communication-related technology that transmits information. Mobile health (mHealth) and Internet of Things (IoT) are emerging keywords in various industries, including healthcare, and are being applied to multiple fields.

Aging is an unavoidable demographic trend worldwide, with the main health problem being frailty. The increase in weakness and chronic diseases of older adults consumes socioeconomic resources in the community, and the increase in medical expenses is a serious problem. Care for vulnerable older adults has often been undertaken in medical and nursing facilities, but many recent studies have shown that providing healthcare in a familiar home environment has the same or more effective clinical outcomes compared to care in medical facilities (1–3). In particular, the perceived quality of life increases when older adults live in their own homes. The fact that they can continue to be in an environment they are familiar with gives them psychological stability, which can have a positive effect on their mental and social health.

From the service receiver's perspective, there is no need for older adults to wait until formal care services become available. This reduces waiting time and increases the participant's ability to self-manage, which can have great long-term health effects. Ultimately, the goal of the healthcare service using ICTs is to serve as a self-management mechanism that can be intuitively used by participants without requiring special effort by health professionals (1, 2). In the past, to evaluate the physical activity of older adults, one had to use a pedometer and write evaluation notes in a notebook. However, when ICT is used, the number of steps taken and calories consumed are automatically measured by the smartphones that are linked to the database, so that health experts can check and manage it in real time. The purpose of using ICT for health management is to incorporate technology into the life of the user, thus helping them efficiently manage their health with minimal effort.

ICT provides an advantage for service providers in that one health professional can manage more people simultaneously, thereby increasing work efficiency and reducing costs. The time for providing indirect services such as data collection and preparation for patient visits is greatly reduced, which allows health professionals to focus more on direct healthcare services. In terms of efficiency and effectiveness, ICT healthcare services targeted towards older adults are an approach with great potential (1).

Representative projects that aimed to prevent the frailty of older adults using ICT are PERSSILAA (Personalized ICT Supported Service for Independent Living and Active Aging), SPRINTT, and My-AHA (4, 5). They were implemented to prevent weakness in older adults in the community and to achieve independent and successful aging. Cognitive, nutrition, and exercise programs were conducted after primary screening and secondary detailed evaluation in groups and individually. Technologies such as video calls, messages, remote monitoring, and health measurement through smartphone apps were grafted. These projects yielded positive results in improving activities of daily living, quality of life, and frailty. A previous study confirmed that healthcare services using ICT had a positive effect on exercise ability, cognitive function, and depression (4, 6). Smart speakers have different effects depending on the participants’ attitudes toward smart devices and gender. They are also easy to use because they operate as a voice interface and can have a positive effect on emotion through a conversation function (6, 7).

Home care services are implemented at 254 public health centers across South Korea as part of a community-wide health promotion project, in which a nurse visits the homes of those aged 65 or older, periodically checking health status and counselling. However, from 2020 to 2022, it was difficult to manage health through direct home visits due to the spread of COVID-19 in the community. To solve this problem, a pilot project, known as the “AI-IoT Healthcare Service for Older Adults,” has been implemented since 2020 by the Ministry of Health and Welfare, the Korea Health Promotion and Development Institute, and Korea Social Security Information Services to convert healthcare visits for the vulnerable from face-to-face to virtual visits.

This study statistically verifies the effect of the local health center's newly attempted “AI-IoT healthcare service for older adults” in Seoul, South Korea. The pilot project consisted of providing wearable devices to older adults living in the local area, along with a smartphone app that can check healthcare missions and monitor this information to offer non-face-to-face professional counseling with exercise experts, nutritionists, and home care nurses.

According to the theory of planned behavior, attitudes, subjective norms, and perceived behavioral control influence intentions. Further, behavioral intentions are strongly correlated with behaviors. Attitudes toward health behaviors are determined by beliefs about health outcomes and evaluations of the values associated with those outcomes (8). Health interventions using ICT can increase value and a perceived sense of control over health outcomes. Positive health results were expected in the group that was additionally provided the smart speaker, due to higher adherence to health behaviors than the group that used the App alone. Previous studies found that the communication function of smart speakers, music, and ASMR functions had a positive effect on relieving depression (7, 9). Previous research has also shown that healthcare services using ICTs are an effective approach for the successful aging of community-dwelling older adults, but it is necessary to accumulate more knowledge about the acceptability and effectiveness of the various types of interventions. Therefore, this study discerns the effect of ICT healthcare services for older adults on depression and health behaviors (10).

The purpose of this study is (1) to identify the effect of health management services using healthcare apps and smart speakers with older adults in the community on health behavior, health status, and depression, and (2) to compare the effects on health status, health behavior, and depression between the group that was provided both the healthcare app and the smart speaker and the group that was only provided the healthcare app.

## 2. Methods

### 2.1. Study design

This study consisted of a nonequivalent control group pretest-posttest design. The participants were either assigned to the experimental group [i.e., the smart speaker group (SS)], who were provided with a smartphone app (“Health Today”), wearable devices, and smart speakers, or to the control group, that only received a smartphone app and wearable devices [i.e., the healthcare app group (HA)].

Older adults registered with the health center home care service who agreed to participate in the study but did not agree to the provision of smart speakers were assigned to the control (HA) group. Those who agreed to use the smart speaker were assigned to the SS group, in accordance with the Korean national project guidelines for those who live alone, have low social contact, or experience depression. The recruitment of study participants started in July 2021 after the IRB approval date, and the preliminary evaluation was completed by August 2021. The post evaluation was conducted between December 2021 and January 2022.

### 2.2. Participants

Our participant sample consisted of older adults aged 65 or older registered for the home care services provided by a public health center in Seoul. The criteria for the selection of study participants were the ability to maintain cognitive function to use IoT devices and to understand the survey or follow the instructions of the visiting nurse. Chronic diseases such as hypertension, diabetes, cancer, hyperlipidemia, cerebrovascular disease, and cardiovascular disease may be present in the participants. The exclusion criteria for participation in the study were those diagnosed with dementia or significantly reduced cognitive function who were unable to complete questionnaires and follow the visiting nurse's instructions. People who had taken drugs or been diagnosed by a doctor for alcoholism, depression, schizophrenia, or any other type of psychosis were also excluded. If it was determined that it was impossible for a participant to continue participating in the study due to a deterioration of health during the study or if they passed away, they were excluded. Further, if voluntary participation was difficult to guarantee, or if participants wanted to withdraw their participation, these individuals were removed from the study.

### 2.3. Ethical consideration

The entire process of this study was planned after deliberation by the Public Institutional Review Board of the Korea National Institute for Bioethics Policy, and the study termination report was completed in compliance with the ethical guidelines (Public IRB No. 2021-0808-004). Recruitment and pre- and post-evaluation of the control and intervention groups were conducted at public health centers. Participants provided consent after the lead researcher explained the research at the time of registration and pre-evaluation. The participants also received a separate explanation through written consent forms.

### 2.4. Interventions

Following a booking for a visit to the public health center, the participants underwent a multicomponent intervention which included a consent form, pre-evaluation, 6 months of non-face-to-face health counseling, and health management information for using ICT devices. At the end of the 6-month service, the same items were subject to a post-evaluation. All the participants received non-face-to-face health counseling at least once a month. The healthcare missions consisted of the following: eating 3 meals per day, walking 5,000 steps or 30 min per day, taking prescribed medication on time, going outside at least once a day, measuring blood pressure once a day if participants had hypertension, measuring glucose level regularly if participants had hyperglycemia and drinking 8 cups of water per day (see Supplementary Figure S1). The participants connected their health data (step count, blood pressure, blood glucose, healthcare mission) to the smartphone app through wearable devices in real-time. This information was remotely monitored by visiting nurses, exercise experts, nutritionists, and other experts from the health center. Non-face-to-face consultations were conducted more than once based on this information. Health education materials were also provided in a non-face-to-face manner, and pictures or video links related to healthcare were sent to the participants’ mobile phones at least once a month. Using the app's push notifications, we sent a text message encouraging the participants to perform a healthcare mission at least once a week. The home care nurses monitored blood pressure, blood glucose levels, and step count levels at least once a week and provided consultations if there were any abnormalities. Table 1 presents the functions of smart speakers, smartphone apps, and wearable devices provided for each group.

**Table 1:** Functions of healthcare devices by intervention groups.

Provided devices		Healthcare app + smart speaker user group (SS group)	Healthcare app user group (HA group)
Smart speaker	Emotional support	Tactile or voice recognition type conversation	None
	Disease management	Chronic disease related medication notification	
	Safety management	Sending voice rescue messages: 24/7 monitoring in conjunction with security companies	
	Cognitive function	Cognitive Enhancement Quiz Program	
	Life information	– Various information necessary for senior life (living information, health information provided) – Wakeup call	
	Exercise	Provide gymnastics program according to voice guidance	
	Sound contents	News, music, radio, religious content, ASMR (sound of waves, wind, etc.)	
Smartphone app (“Health Today”^a^)	– Monthly healthcare mission assignment (e.g., “Eat three meals a day,” “Walk more than 5,000 steps every day,” etc.) – Remote consultations with exercise therapists, nutritionists, and visiting nurses – Send health-related text messages and push notifications on the smartphone
Wearable devices	Wrist-worn activity monitor, Bluetooth blood pressure monitor, Bluetooth blood glucose monitor, Bluetooth scale (Health condition monitored in conjunction with the smartphone app)

ASMR, autonomous sensory meridian response; SS, App + smart speaker; HA, healthcare app.

^a^ Smartphone healthcare app, which is developed by the Korean Health Promotion Institute.

### 2.5. Instruments

#### 2.5.1. General characteristics

The factors reported to potentially influence depression and health behavior, such as sex, age, family type (living alone, couple of older adults, multicultural families, etc.) were investigated.

#### 2.5.2. Physical measurements and health status

• Body mass index (BMI): BMI is calculated as weight (kg)/height (m^2^). The participants were classified as normal (18.5 ≤ BMI < 25 kg/m^2^), underweight (<18.5 kg/m^2^), or obese (≥25 kg/m^2^).
• Relative hand grip strength [Absolute grip strength (kg)/weight (kg)*100, %]: grip strength was measured using a hand dynamometer by trained nurses. The arm is naturally lowered to the research participant, and both hands are measured alternately twice. The participants were asked to hold the grip dynamometer, contract it with maximum force for 5–10 s, and then record the maximum value out of measurements. Relative grip strength was judged according to “normal,” “risk,” and “weak” stages by referring to the standard value according to the age and gender of the participant (11, 12).
• One leg balance test: After having the elderly stand on one foot, the number of seconds they can stand is measured with a stopwatch. If it is less than 5 s, it is evaluated as abnormal, and if it is more than 5 s, it is evaluated as normal (13).
• Five-time-seat-to-stand test (FTSTS): The FTSTS score measures the time it takes the participants to transfer from a sitting position to a standing position and back to a sitting position 5 times. The age matched norms scores are 11.4 s for the 60–69 years age group and 12.6 and 14.8 s for the 70–79 and 80–89 years age group, respectively (14).
• Diagnosis of chronic disease (high blood pressure, diabetes, stroke, cancer, arthritis, other diseases).

#### 2.5.3. Health behavior

• Dietary diversity scores (DDS): The daily intake of cereals, proteins, vegetables, fruits, milk, and dairy products is given as 1 point for each group and 0 points for non-intake, ranging from 0 to 5 points. The higher the score, the more balanced the food consumption.
• Physical activity: Average exercise frequency per week. The number of days of moderate-intensity exercise was investigated through a self-response, ranging from 0 to 7 days.

#### 2.5.4. Exercise self-efficacy

The exercise self-efficacy tool, developed by Marcus et al. (15) and translated by Lee and Jang (16), was used with a total of 5 items. This tool uses a 5-point scale that assesses confidence in one's ability to consistently perform exercise in any situation. The higher the value, the higher the self-efficacy, with 1 point for “not at all confident” and 5 points for “very confident,” and a higher score indicating a higher sense of self-efficacy. Total scores range from 5 to 25.

#### 2.5.5. Depression

The Geriatric Depression Scale (GDS) was developed with 30 items. Sheikh and Yesavage (17) developed a short form version consisting of 15 items based on the diagnostic validity study on GDS. In Cho et al.'s study (18), the validity of the Korean version of the GDS in short form was verified and the reliability was 0.88. It consists of a total of 15 items and measures “yes” and “no” on a binary scale for each symptom. A higher score indicates a higher level of depression. If the cut-off point is 8 or higher, it reveals a risk of depression.

#### 2.5.6. Data analysis

**Figure 1:**
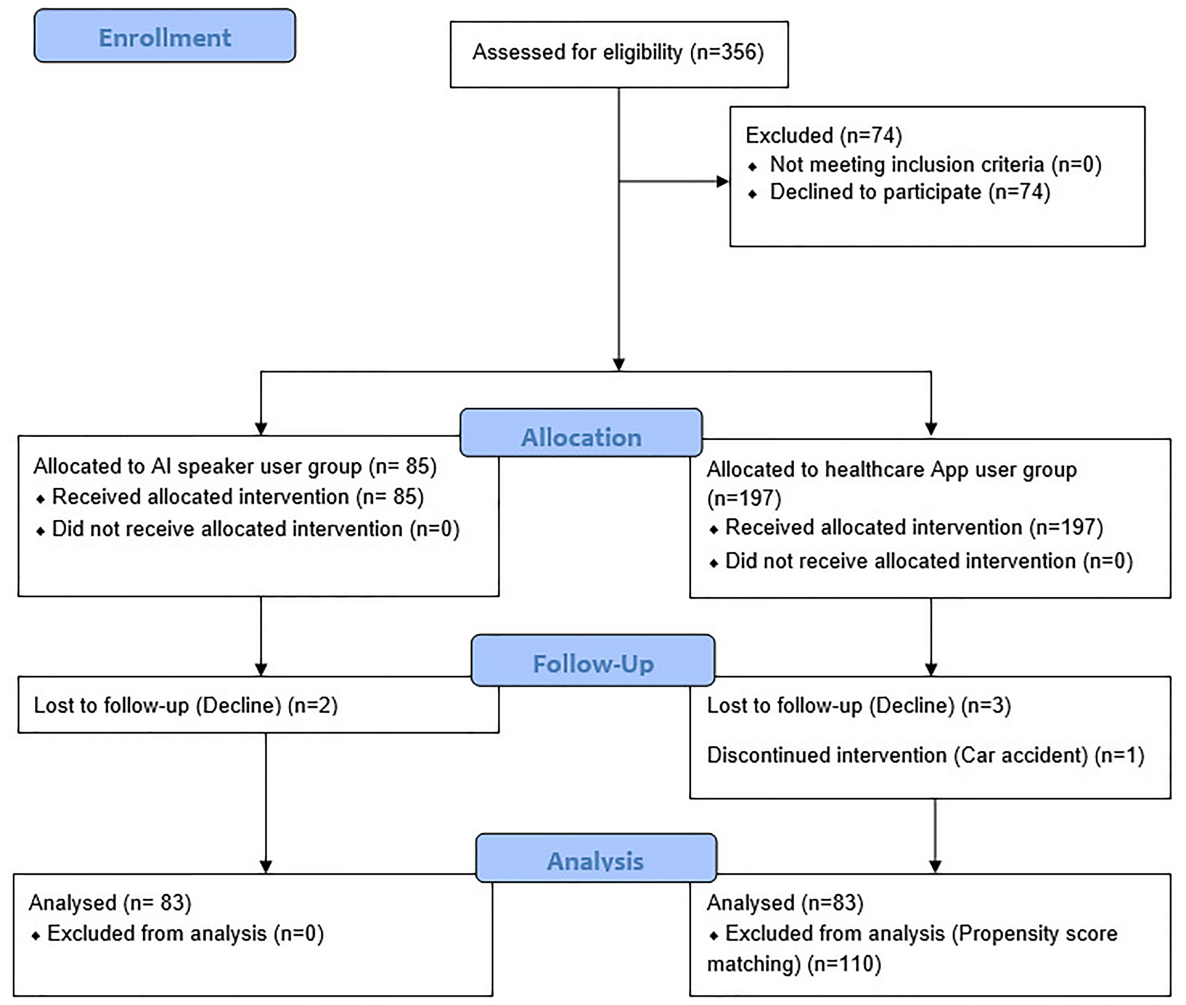
CONSORT flow diagram of the study.

Of the total of 356 participants, those who lived alone, had low social contact, or were depressed were assigned to the SS group, according to the health center project guidelines. 74 out of 356 people refused to participate in the study. 85 people were assigned to the SS group and 197 people to the HA group. Two people in the SS group and four people in the HA group dropped out due to an accident or because they withdrew from the study. A propensity score matching (PSM) method using depression scores was used to accurately analyze the effects of the SS group and the HA group. The propensity score was calculated by performing logistic regression analysis, with age and depression as the independent variables, and the provision of a smart speaker as the dependent variable. Participants with similar scores were matched 1:1 between the two groups; 83 people who used the healthcare app, and 83 people who used both the app and the smart speaker were matched and included in the final analysis (Figure 1).

The Shapiro-Wilk normality test was performed on the data; a *t*-test and the Wilcoxon rank sum test were performed on the continuous variables; the Chi-squared test and Fisher's exact test were performed on the categorical variables as appropriate methods, according to the normality results. The homogeneity of the pre-screening by group was verified for the analysis of the intervention effect. For continuous variables, the Wilcoxon rank sum test or *t*-test for differences in pre-post values was used, and for qualitative variables, Fisher's exact test or $\chi^{2}$ test was used. Statistical analysis was performed using Stata 17.0 (19). Statistical significance was based on an alpha value of 0.05.

## 3. Results

### 3.1. Participants’ general characteristics

Table 2 presents the general characteristics of the study participants: 24.1% were male and 75.9% were female, and both groups showed no statistical difference. The mean age was 71.05 ± 4.65 years, and there was no statistical difference between the two groups. Among those living alone, 55 were from the SS group (66.27%), and 36 (43.37%) were from the HA group. This study was conducted as part of a public health center project; these results were produced according to the standard for distributing smart speakers to people living alone, and it is therefore necessary to pay attention to the interpretation of the results.

**Table 2:** General characteristics and health status of participants.

Category		Total	HA	SS	*t* or $\chi^{2}$	*p*
		(*N* = 166)	(*N* = 83)	(*N* = 83)		
		*n* (%) or mean ± SD				
Sex	Male	40 (24.1)	20 (24.1)	20 (24.1)	0.00	>0.99
	Female	126 (75.9)	63 (75.9)	63 (75.9)		
Age	71.05 ± 4.65	70.37 ± 4.83	71.73 ± 4.4	1.90	0.059
Characteristics of family	Multicultural family	1 (0.6)	0 (0)	1 (1.2)	12.686	0.027^*^
	Living with grandchildren	1 (0.6)	1 (1.2)	0 (0)		
	Living alone	91 (54.82)	36 (43.37)	55 (66.27)		
	Living with spouse	46 (27.71)	31 (37.35)	15 (18.07)		
	Living with children	27 (15.72)	15 (18.07)	12 (14.46)		
Hypertension	No	72 (43.37)	39 (46.99)	33 (39.76)	0.88	0.347
	Yes	94 (56.63)	44 (53.01)	50 (60.24)		
Diabetes	No	123 (74.1)	65 (78.31)	58 (69.88)	1.53	0.215
	Yes	43 (25.9)	18 (21.69)	25 (30.12)		
Stroke	No	157 (94.58)	79 (95.18)	78 (93.98)	0.12	0.732
	Yes	9 (5.42)	4 (4.82)	5 (6.02)		
Cancer	No	152 (91.57)	80 (96.39)	72 (86.75)	4.99	0.026^*^
	Yes	14 (8.43)	3 (3.61)	11 (13.25)		
Arthritis	No	120 (72.29)	64 (77.11)	56 (67.47)	1.91	0.167
	Yes	46 (27.71)	19 (22.89)	27 (32.53)		
Hyperlipidemia	No	113 (68.07)	62 (74.7)	51 (61.45)	3.353	0.067
	Yes	53 (31.93)	21 (25.3)	32 (38.55)		
Number of chronic diseases	0	31 (18.67)	21 (25.3)	10 (12.05)	8.575	0.0726
	1	57 (34.34)	31 (37.35)	26 (31.33)		
	2	40 (24.1)	18 (21.69)	22 (26.51)		
	3	30 (18.07)	10 (12.05)	20 (24.1)		
	4	8 (4.82)	3 (3.61)	5 (6.02)		

HA, healthcare app user group; SS, app + smart speaker user group.

* *p* < 0.05.

**p < 0.01.

***p < 0.001.

A total of 94 (56.63%) out of the 166 participants had hypertension, 43 (25.9%) had diabetes, 9 (5.42%) had a stroke, 14 (8.43%) had cancer, 46 (27.71%) had arthritis, and 53 (31.93%) had dyslipidemia. Those without chronic disease accounted for 18.67% of the total participants, compared to those with one or more disease at 81.33%.

A BMI of 25 kg/m^2^ or more was considered as obese and less than 18.5 kg/m^2^ as underweight; hence, 63 (37.95%) of participants were obese and 8 (4.82%) were underweight. For the relative grip strength, 32 (19.28%) of the participants were at the risk level and 53 (31.93%) were rated as weak, with no significant difference between the two groups. Ten (10.84%) participants were evaluated as weak in the one leg balance test, and 45 (25.45%) were assessed as weak in the five-times-sit-to-stand test, with no significant difference between the two groups.

### 3.2. Effects of smart speaker and healthcare app on health behavior and depression

Table 3 presents the results of comparison between pre- and post-values of the participants. The level of depression increased in both groups in the post-test compared to the pre-test, and there was no difference in the degree of increase in the post-test between the two groups (HA: 0.83 ± 3.77 and SS: 1.73 ± 3.38, *p *> 0.05).

**Table 3:** Pre-post effects evaluation between the Smart Speaker group and healthcare app user group.

Category		Pre-test			*t* or $\chi^{2}$	*p*	Post-test *n* (%) or *d* (Post-pre)			*t* or $\chi^{2}$	*p*
		Total	HA	SS			Total	HA	SS		
		(*N* = 166)	(*N* = 83)	(*N* = 83)			(*N* = 166)	(*N* = 83)	(*N* = 83)		
GDS	3.5 ± 3.19	3.18 ± 3.05	3.82 ± 3.32	1.276	0.204	1.27 ± 3.6	0.83 ± 3.77	1.73 ± 3.38	1.589	0.114
Exercise self-efficacy	18.47 ± 4.37	18.55 ± 4.56	18.39 ± 4.2	−0.248	0.805	0.58 ± 3.68	0.92 ± 3.61	0.25 ± 3.73	−1.162	0.247
Moderate exercise frequency/week	0	76 (45.78%)	34 (40.96%)	42 (50.6%)	3.893	0.143	57 (34.34%)	29 (34.94%)	28 (33.73%)	0.361	0.835
	1–2	12 (7.23%)	9 (10.84%)	3 (3.61%)			12 (7.23%)	5 (6.02%)	7 (8.43%)		
	Over 3 times	78 (46.99%)	40 (48.19%)	38 (45.78%)			97 (58.43%)	49 (59.04%)	48 (57.83%)		
DDS	0–2	26 (15.66%)	11 (13.25%)	15 (18.07%)	0.949	0.622	23 (13.86%)	12 (14.46%)	11 (13.25%)	6.130	0.047^*^
	3–4	92 (55.42%)	46 (55.42%)	46 (55.42%)			87 (52.41%)	36 (43.37%)	51 (61.45%)		
	5	48 (28.92%)	26 (31.33%)	22 (26.51%)			56 (33.73%)	35 (42.17%)	21 (25.3%)		
BMI	Normal	95 (57.23%)	51 (61.45%)	44 (53.01%)	1.294	0.524	99 (59.64%)	53 (63.86%)	46 (55.42%)	1.227	0.542
	Underweight	8 (4.82%)	4 (4.82%)	4 (4.82%)			9 (5.42%)	4 (4.82%)	5 (6.02%)		
	Obesity	63 (37.95%)	28 (33.73%)	35 (42.17%)			58 (34.94%)	26 (31.33%)	32 (38.55%)		
Relative hand grip strength	Normal	80 (48.19%)	40 (48.19%)	40 (48.19%)	1.138	0.767	94 (56.63%)	48 (57.83%)	46 (55.42%)	0.568	0.753
	Risk	32 (19.28%)	15 (18.07%)	17 (20.48%)			32 (19.28%)	17 (20.48%)	15 (18.07%)		
	Weak	53 (31.93%)	27 (32.53%)	26 (31.33%)			40 (24.1%)	18 (21.69%)	22 (26.51%)		
	Missing	1 (0.6%)	1 (1.2%)	0 (0%)			0 (0)	0 (0)	0 (0)		
One leg balance test	Normal	148 (89.16%)	76 (91.57%)	72 (86.75%)	0.997	0.318	150 (90.36%)	77 (92.77%)	73 (87.95%)	1.107	0.293
	Weak	18 (10.84%)	7 (8.43%)	11 (13.25%)			16 (9.64%)	6 (7.23%)	10 (12.05%)		
FTSTS	Normal	123 (74.55%)	63 (76.83%)	60 (72.29%)	0.448	0.503	137 (82.53%)	70 (84.34%)	67 (80.72%)	1.510	0.219
	Weak	42 (25.45%)	19 (23.17%)	23 (27.71%)			24 (14.46%)	9 (10.84%)	15 (18.07%)		
	Missing	0 (0)	0 (0)	0 (0)			5 (3.01%)	4 (4.82%)	1 (1.2%)		

HA, healthcare app user group; SS, app + smart speaker user group; BMI, body mass index; FTSTS, five-times-sit-to-stand test; GDS, geriatric depression scale; DDS, dietary diversity scale.

* *p* < 0.05.

**p < 0.01.

***p < 0.001.

The average value of exercise on self-efficacy in the pre-test was 18.55 ± 4.56 in the HA group and 18.39 ± 4.2 in the SS group. There was no difference between the posttest and pretest (d) between the two groups (HA: 0.92 ± 3.61 and SS: 0.25 ± 3.73, *p *> 0.05). The proportion of those who did not exercise at all was 45.78% (76 out of 166 participants) and decreased to 57 (34.34%) after 6 months of intervention. However, there was no difference between the HA and SS groups ($\chi^{2}$ = 3.893, *p *> 0.05).

26 people (15.66%) had a dietary diversity score between 0 and 2. After 6 months, the number decreased to 23 (13.86%). In addition, the proportion of those who ate from all five food groups evenly increased from 48 (28.92%) to 56 (33.73%). Although the pre-test values were the same, there was a statistically significant difference between the two groups in the post-test. In the HA group, the number increased from 31.33% to 42.17%, but in the SS group, it decreased from 26.51% to 25.3%. However, in the SS group, those who consumed 0–2 food groups decreased from 18.07% to 13.25%, and those who consumed 3 or 4 food groups increased from 55.42% to 61.45%, thus indicating a positive change.

In the case of BMI, 95 people (57.23%) were evaluated as normal at the pre-evaluation, and 99 (59.64%) at the post-evaluation, a similar level. The obese group also remained at a similar level: 63 (37.95%) at the pre-evaluation compared to 58 (34.94%) at the post-evaluation. There was no statistically significant difference between the HA group and the SS group for both pre and post values.

In terms of relative handgrip strength, the number of those evaluated as normal increased from 80 (48.19%) to 94 (56.63%), and the pre- and post-evaluation were the same for those evaluated as at-risk at 32 (19.28%). The number of those evaluated as weak decreased from 53 (31.93%) in the pre-evaluation to 40 (24.1%) in the post-evaluation. There was no statistically significant difference between the HA and SS groups ($\chi^{2}$ = 0.568, *p *> 0.05).

As a result of the one leg balance test, the normal group increased slightly from 148 (89.16%) to 150 (90.36%), but no significant difference was found between the HA and SS groups ($\chi^{2}$ = 1.107, *p *> 0.05).

Those who were evaluated as normal during the FTSTS test increased from 123 (74.55%) in the pre-evaluation to 137 (82.53%) in the post-evaluation. However, there was no significant difference between the two groups ($\chi^{2}$ = 1.510, *p *> 0.05).

## 4. Discussion

Owing to the recent COVID-19 pandemic, the introduction of non-face-to-face healthcare has accelerated. In South Korea, the introduction of ICT healthcare services is progressing rapidly with the full support of the government, not only in private medicine but also in the public health field. To integrate digital technology as one of the health management methods, significant economic, time, and human resources are being mobilized. However, rather than the indiscriminate introduction of the digital method, it is time to determine what specific function of digital devices to provide to the target population and to accurately verify its effectiveness.

The first important finding of this study is that both the HA and SS group showed positive health status and behavior changes at the time of post-evaluation. Though older adults with low digital literacy should first be educated on these technologies, our study demonstrated that there were significant improvements in health behaviors after adaptation to digital devices. As Table 3 reveals, perceived self-efficacy in exercise, moderate exercise frequency per week, the diet diversity scale, relative grip strength, and FTSTS were positively changed during post-evaluation compared to the pre-evaluation. On the contrary, depression increased, and BMI and balance test scores were maintained at similar levels in both groups after 6 months.

According to a systematic review, studies have found that IoT-enabled health care services can lead to improved outcomes in health and wellbeing for older adults, such as improved medication adherence, better management of chronic conditions, and improved quality of life (20). Similar to this study, previous studies also showed a statistically significant increase in exercise frequency and improved eating habits (21–24). Recording eating habits and exercise frequency with mobile apps and wearable devices can raise the level of awareness of health behaviors, making it easier for older people to change their behavior, rather than just counseling them to change. In the study of Barnason andZimmerman (21), 36 telephone counseling sessions were conducted intensively over 3 months, during which self-efficacy improved as in the results of this study. However, Fukuoka and Gay (23) reported no improvement in self-efficacy after providing a multicomponent intervention using a mobile app and wearable device for 5 months. Unlike many previous studies that showed significant improvements in BMI and weight loss, this study showed similar levels after 6 months (21, 23, 25). In addition, among objective indicators such as BMI, relative grip strength, balance test, and FTSTS test levels were classified according to risk level, so there may not have been a significant difference across categories during the 6-month study period. There were no previous studies using FTSTS, relative grip strength, balance test, etc., which are important indicators for predicting frailty.

Second, unlike previous studies, our hypothesis that listening to songs, Autonomous Sensory Meridian Response (ASMR), and conversation functions (which are the main functions of smart speakers) will have a positive effect on reducing depression has not been supported (26). Rather, the feeling of depression increased further in the post-test, which may be due to the influence of the Corona-blue due to COVID-19, an external environmental factor, or the test-retest bias. In addition, as there was no statistically significant difference in the degree of increase in depression between the two groups, it was not possible to reveal any additional benefits of the smart speakers on depression in older adults living alone or those who were socially frail. According to the results of a recent study, depression and loneliness were significantly reduced in older adults after 2 months of using the same smart speaker used in our study. However, no statistically significant difference was found between those who frequently used smart speakers and those who used them intermittently. Therefore, it is difficult to infer that depression decreased due to the direct influence of smart speakers (9).

There are studies that show a statistically significant reduction in depression when a smart speaker in the form of a child doll is provided to an elderly person with type 2 diabetes and cognitive decline who lives alone (7, 27, 28). In addition, in a path analysis of the effects of smart speakers on health behavior and depression, it was found that health behavior mediates attitudes toward smart speakers, leading to an alleviation of depression (29). Only older adults who had a positive attitude toward a smart speaker showed a significant effect.

A limitation of this study is that it was not possible to control attitudes and usage patterns toward smart speakers. Follow-up studies should aim to identify and control usage patterns for digital devices. Since most previous studies applying eHealth or mHealth were for middle-aged people, more research on mHealth-related effects in older adults seems necessary (20). Variables such as relative grip strength, balance test, and FTSTS, which are mentioned as reliable tools to predict frailty, should be considered.

The importance of chronic disease management for older adults is further emphasized when considering the threat of infectious diseases and mortality statistics (30, 31). Another problem is that vulnerable older adults, who are the main target group of public health, may have difficulty managing their diseases due to fear of visiting public health centers, hospitals, and clinics, and due to concerns regarding social distancing. For public health centers to achieve chronic disease management and health promotion in addition to their role in quarantine, it is necessary to expand non-face-to-face healthcare and attempt to effectively integrate it with existing services.

Despite the advantages of international trends and previous research results, the reason that ICT has not been widely used as a health promotion intervention for older adults is that there are doubts about its acceptability and effectiveness from health professionals and older adults (32). Since qualitative research still dominates the research field of ICT healthcare services, many experimental studies need to be conducted (33).

Health management results may differ depending on the ability to use smartphones and the IoT, and the ability to acquire, understand, and utilize health information. Therefore, if the device or application used by older adults is not developed so that the user can intuitively use it, it can become a barrier to health management. Although the acceptance of ICT by older adults is still lower than that of adults, it is gradually gaining acceptance among the former (34). Hence, active intervention by health experts is important, which is why accessible technology for older adults needs to be further developed (35).

## 5. Conclusion and recommendations

ICT can be sufficiently attempted even for older adults as it is an efficient healthcare approach that can be used more actively as the older adult population becomes increasingly accustomed to digital devices. In addition, as older adults in the community cannot be managed by medical staff near them, such as in hospitals, ICT is a useful method to monitor symptoms of chronic diseases, detect abnormalities, and manage health behaviors remotely, and it will be an important leap forward for the healthcare industry. However, to provide an ICT device for the health management of socially and economically vulnerable older adults in the public domain, such devices should be introduced after verifying the older adult-friendly interface and functions of the healthcare device and ascertaining whether it meets the purpose and goal set by the national health project.

## Data Availability

The datasets presented in this article are not readily available because this study was conducted at a public health center in South Korea, and disclosure of data other than for research purposes is not permitted. Requests to access the datasets should be directed to Dasom Kim, dudurdaram@naver.com.
