# Supplementary material for: Can healthcare apps and smart speakers improve the health behavior and depression of older adults? A quasi-experimental study
Source: Front Digit Health. 2023 Feb 23;5:1117280. doi: 10.3389/fdgth.2023.1117280 (PMC9996178; doi:10.3389/fdgth.2023.1117280)
Supplement: Supplementary file 1 [file Table1.docx]

Supplementary Material

# Supplementary Figure

| Confirmation of consultation schedule | | |
| --- | --- | --- |
| ↓ | | |
| Referral to nutritionist and exercise specialist, if necessary | | |
| ↓ | | |
| Assessment of medical history and chief complaints  Physical measurements and pre-evaluation questionnaires | | |
| ↓ | | |
| Motivational evaluation (behavioral change stage) | | |
| ↓ | | |
|  | ⇨ | <Nursing Consultation>  ① Blood pressure and blood sugar self-measurement management  ② Monitoring of abnormal values  ③ Medication guidance, lifestyle management (non-smoking, abstaining from alcohol)  ④ Recommendation for regular check-ups  ⑤ Providing educational materials for each disease |
| Nursing, physical activity, and nutrition counseling  Principle 1. Identify the needs of the target audience  Principle 2. Apply counseling techniques according to the stage of behavior change  Principle 3. Customize counseling according to health problems by chronic disease (hypertension, diabetes, dyslipidemia, etc.) |  |  |
|  | ⇨ | <Nutrition Consultation>  ① Diagnosis of eating habits: 24-hour recall method, problem diagnosis  ② Establishment of customized management plan and provision of educational materials  ③ Provision of diet for each chronic disease |
|  |  |  |
|  | ⇨ | <Exercise consultation>   1. Diagnosis of physical activity level (METs: Metabolic equivalents) 2. Establish a physical activity plan 3. Provide customized exercise video (using YouTube channel) 4. IoT device utilization: smart band pulse rate   - Increase exercise intensity by suggesting target heart rate  * Maximum heart rate formula: 220-age, target heart rate = (1)+ resting heart rate |
| ↓ | | |
| Set the next consultation date | | |

**Supplementary Figure 1.** Intervention process for all participants.
